# SOX13/TRIM11/YAP axis promotes the proliferation, migration and chemoresistance of anaplastic thyroid cancer

**DOI:** 10.7150/ijbs.54194

**Published:** 2021-01-01

**Authors:** Jianing Tang, Zelin Tian, Xing Liao, Gaosong Wu

**Affiliations:** Department of Thyroid and Breast Surgery, Zhongnan Hospital of Wuhan University, Wuhan, China.

**Keywords:** anaplastic thyroid cancer, TRIM11, YAP, stabilization, mono-ubiquitination

## Abstract

Anaplastic thyroid cancer (ATC) is one of the most aggressive and virulent solid tumors. The ubiquitin proteasome system presents in all eukaryotic cells and is essential for cellular homeostasis. While its underlying role in ATC remains largely unclear. TRIM11 is an E3 ubiquitin ligase and has been reported to act as an oncogene in several human cancers. The present study aims to reveal the oncogenic function of TRIM11 in ATC. Western blot was used to measure the protein expression of TRIM11 and YAP, while the YAP target genes were measured by real-time PCR. CCK8 assay was used to detect cell viability; wound-healing assay and transwell assay were used to measure the migration ability of ATC. The xeno-graft tumor model was used for *in vivo* study. Immuno-precipitation assay was used to detect the interaction domain between YAP and TRIM11. And the ubiquitin-based Immuno-precipitation assays were used to detect the specific ubiquitination manner happened on YAP. TRIM11 depletion significantly decreases cell proliferation and migration capabilities of ATC cells, and elevates cell sensitivity to chemotherapy, which effect could be further rescued by YAP overexpression. TRIM11 depletion decreases YAP protein level and YAP/TEAD target genes, such as CTGF, ANKRD1 and CYR61 in ATC. Indicating that TRIM11 is a regulator of Hippo signaling pathway. Immuno-precipitation assay shows that the RING domain of TRIM11 is essential for the interaction with WW domain of YAP. Further mechanistic analysis suggests that TRIM11 promotes the mono-ubiquitination of YAP, thus prolongs its protein half. Furthermore, TRIM11 promoter analysis revealed that SOX13 activates TRIM11 transcription by binding to the promoter of TRIM11. In summary, our study describes the oncogenic function of TRIM11 in ATC, which acts as a post-translational modulating factor of Hippo pathway. Targeting TRIM11 may be a potential therapeutic method for ATC treatment.

## Introduction

Thyroid cancer ranks in ninth place in malignancy worldwide. It is the most commonly diagnosed endocrine malignancy, accounting for more than 90% of all endocrine cancer cases 1, 2. Based on the histopathological features and the degree of differentiation, thyroid cancer has been divided into three major categories: well-differentiated thyroid cancer (WDTC) which includes papillary thyroid cancer and follicular thyroid cancer; poorly-differentiated thyroid cancer (PDTC) and undifferentiated/anaplastic thyroid cancer (ATC) 3. Well differentiated thyroid cancer is the most common thyroid cancer, accounting for approximately 90% of all cases. It is one of the most indolent tumors, which exhibits excellent prognosis with > 98% 5-year survival 4-6. ATC is a small subset of thyroid cancer. It accounts for only 2-5% of thyroid cancer 7-9. Due to the high proliferation rate and invasive behavior of ATC, it is responsible for 40-50% of total thyroid cancer-related deaths. Median survival of ATC patients is 3 to 5 months. 1-year survival rate is estimated at 10-20% 10-12. ATCs rarely respond to the conventional treatment such as radioactive iodine and chemotherapy, and treatment options of ATC remain limited and largely ineffective 13.

Ubiquitination is an important posttranslational modification, which is a central component of the cellular protein-degradation machinery and essential for cellular homeostasis 14. The ubiquitin proteasome pathway (UPP) presents in all eukaryotic cells. It is an extensive and complex protein degradation pathway and regulates various biological processes such as cell survival, apoptosis, DNA repair, cell-cycle progression, signal transduction, antigen presentation and protein turnover by the proteasome 15-17. Ubiquitination involves the sequential transfer of an ubiquitin molecule mediated by three enzymes: ubiquitin-activating enzyme (E1), an ubiquitin conjugating enzyme (E2) and an ubiquitin ligase (E3) 18. E3s are involved in many cellular processes and various types of cancer. They act as oncogenes or tumor suppressors according to the nature of their substrates via regulating protein stability and subcellular localization 19. However, the underlying role of E3 ligase in thyroid cancer, especially in ATC remains largely unclear.

Substrates may be ubiquitinated as mono-ubiquitination, multiubiquitination (mono-ubiquitination of several residues), or polyubiquitination. Polyubiquitination chain can be linked at seven lysine positions (6, 11, 27, 29, 33, 48, and 63) of ubiquitin 20. These modifications may result in different cellular outcomes. Mono-ubiquitination can regulate processes such as DNA repair, gene expression, endocytosis, and protein stability 21-23. Multi-ubiquitination is associated with receptor endocytosis 23. K11- or K48-linked polyubiquitination generally leads to proteasomal degradation 24, whereas K63-linked ubiquitin chains can function in signaling and endocytosis^21^. The role of K6-, K27-, K29-, and K33-linked ubiquitin chains are less clear. Accumulating studies have reported that mono-ubiquitination and polyubiquitination compete to modify the substrate protein to regulate its stability, and mono-ubiquitination is more likely to confer the substrate stability by inhibiting its polyubiquitination and degradation 25, 26.

TRIM11 is an E3 ubiquitin ligase and belongs to the tripartite motif (TRIM) protein family. The oncogenic function of TRIM11 has been investigated in different types of human cancers, including hepatocellular carcinoma cancer, lung cancer, and glioma 27-30. In our present study, we observed that TRIM11 was associated with more aggressive phenotypes of ATC. Mechanistic studies revealed that TRIM11 could enhance YAP protein stability. Targeting TRIM11 could be a promising therapeutic approach for ATC treatment.

## Materials and methods

### Cell culture

ATC cell lines (CAL62, and KHM-5M) and HEK293T cells were obtained from the American Type Culture Collection (ATCC; Rockville, MD, USA). All cell lines possessed cell line authentication via Short Tandem Repeat (STR), which is performed through PowerPlex 21 system, turning out that The STR data keep consistent with its in ATCC. HEK293T and CAL62 were maintained in Dulbecco's Modified Eagle's Medium (DMEM, 41965, Life Technologies) supplemented with 10% fetal bovine serum (FBS). KHM-5M cells were cultured in RPMI/1640 medium (42401, Life Technologies) supplemented with 10% FBS.

### Plasmids and RNA inference

We obtained the wild type/mutant constructs of TRIM11 and YAP from Hanbio Biotechnology Co. Ltd. (Shanghai, China). The HA-Ub, -K0, -K6, -K11, -K27, -K29, -K33, -K48, and -K63 plasmids were obtained from Addgene. We transfected the plasmids into cells using Lipofectamin 2000 (1662298, Invitrogen). Small interfering RNAs were used for specific gene knocking-down. The TRIM11 siRNA sequences used were: siRNA #1: 5'- GAGAGUAGAUGUCCUAUAA -3'; siRNA #2: 5'- GGGUGAGUUCGAGCGUCUU-3'. And YAP siRNA sequences were: siRNA #1: 5'- GCUCAUUCCUCUCCAGCUU-3'; siRNA #2: 5'- CACCUAUCACUCUCGAGAU-3'. SiRNAs were transfected using RNAiMAX reagent (13778150, Invitrogen).

### RNA extraction and qPCR analysis

RNeasy plus mini kit (Qiagen, Germany) was used to extract the total RNA from the cancer cells. PrimeScript RT Master Mix (Takara, Japan) was used to perform reverse transcription. qRT-PCR was performed using the SYBR green mix (Toyobo, Japan) with the CFX96TM Real-time PCR Detection System (Bio-Rad, USA). The 2^-ΔΔCt^ method was used to calculate the relative expression. 36B4 was used for internal control. All assays were performed in triplicates.

### Proliferation, cell cycle and colony formation assay

2×10^3^ CAL62 and KHM-5M were seeded in 96-well plates. The proliferation of cells was measured in three independent experiments using Cell Counting Kit-8 (CCK8) at indicated time points. The cell cycle phases determined by relative DNA content were analyzed by flow cytometer (Beckman, USA). For the clone formation assay, ATC cells were seeded into 6-well plates at a density of 2×10^3^ cells per well. After cultured in complete medium for 2 weeks, Colonies were fixed with 4% paraformaldehyde for 15 min, followed by crystal violet solution staining for 5 min. EdU incorporation assay was performed as our previously described 31.

### Wound-healing assay

ATC cells were seeded into 6-well plates and cultured until full confluent; the cell monolayer was scratched in a straight line using a 200 μl sterile pipette tip and washed with PBS three times. Cells were maintained in serum-free medium and images were captured every 24 hours.

### Transwell migration assay

Migration capability was measured using 24-well transwell chamber systems (Corning, USA) with 8.0 µm pore size. Cells were seeded in the upper chamber insert and cultured in serum-free medium. The bottom chambers were filled with 10% fetal bovine serum medium. After 24 hours, the migrated cells were fixed with 4% paraformaldehyde for 20 min at room temperature and then stained with 1% crystal violet. The migrated cells were counted and photographed in three randomly selected views.

### Luciferase assay

The luciferase activity of YAP-luciferase reporter was measured using the Dual-Luciferase Reporter kit (MA0518, Meilun Biotechnology, China). The YAP-luciferase reporter plasmid was transfected together with Renilla plasmid into ATC cells. After 24 h, luciferase activity was detected.

### Chromatin immunoprecipitation (Chip) assay

Magna ChIP-seq™ Chromatin Immunoprecipitation Kit (Millipore, Billerica, USA) was used for detecting protein-chromatin interactions. Briefly, the cells were fixed with formaldehyde and sonicated, and incubated with the target protein. The cross-linked DNA fragments were then released from the co-precipitated complexes, purified, and amplified by PCR. Sequences of primers amplifying the TRIM11 promoter regions were as follows: Region 1 (R1), sense: 5'- CCCAGGCTGCCCATAGAAAT-3', antisense: 5'- TTTGGGGATCGAACGGGATG-3'; Region 2 (R2), sense: 5'-GTGGGAGGTTTGGGGGACTG'-3'; antisense: 5'-CCTCAAGGGGCCTGGTTGAAA-3'.

### Immunofluorescence assay

CAL62 and KHM-5M cells cultured on sterile glass cover slips in 24-well plates were fixed in 4% paraformaldehyde for 30 minutes at room temperature. 0.2% Triton X-100 were used to permeabilize cells with for 5 min, and cells were blocked with 10% goat serum for 1 h. Primary antibodies against YAP (mouse, Proteintech), and TRIM11 (rat, Proteintech) were incubate at 4 °C overnight. Followed by incubating with Fluorochrome-conjugated secondary antibodies. The images were acquired using a NIKON80i fluorescent microscope.

### Xenograft tumor model

6-week-old BALB/c athymic nude mice (Beijing HFK Bioscience Co., Ltd. in Beijing, China) were used for construction of xenograft mouse model. 1X10^6^ CAL62 cells were injected subcutaneously to each mouse. We measured tumor size every 3 days. After 30 days, the mice were sacrificed and the tumors were weighted and photographed. All animal experiments were approved by ethnic committee of Zhongnan Hospital of Wuhan University.

### Co-immunoprecipitation assay

We extracted total proteins using co-immunoprecipitation (co-IP) lysis buffer (Beyotime, Beijing, China), cell lysis were precleared with rabbit IgG for 2 h and then incubated with anti-TRIM11 (Proteintech, 10851-1-AP) or anti-YAP (Proteintech, 13584-1-AP) antibody overnight as previously described 32. And we used rabbit IgG (Santa Cruz) as the negative control. The immunoprecipitated proteins were collected for Western blot analysis and analyzed using Anti-YAP or Anti-TRIM11 antibody.

### Western blot analysis

The ATC cells were lysed with Radio Immunoprecipitation Assay (Meilun, China) supplemented with protease inhibitors (Sigma-Aldrich, USA). Total protein was separated using SDS-PAGE and transferred to nitrocellulose membranes (GE Healthcare). Primary antibodies were GAPDH (Proteintech, 60004-1-Ig), YAP (Proteintech, 13584-1-AP), TRIM11 (Proteintech, 10851-1-AP), Myc (Proteintech, 60003-2-Ig), HA (Proteintech, 51064-2-AP), Flag (Proteintech, 20543-1-AP), antibodies. The proteins of interest were visualized using an enhanced chemiluminescence (ECL) kit (Meilun, China).

### RNA sequence analysis

The global gene expression analysis (siControl and siTRIM11) was performed by Beijing Genomic Institute (BGI). SRA database was used to deposit the RNA sequence data (www.ncbi.nlm.nih.gov/bioproject/PRJNA609252/).

### Statistical analysis

Differences among groups were compared using student's t test and one-way ANOVA test. All statistical tests were performed with Prism 7.0 (GraphPad, USA). Statistical significance is represented in figures by: *p < 0.05; **p < 0.01, ***p < 0.001.

## Results

### TRIM11 depletion inhibits anaplastic thyroid cancer cell proliferation and migration

**Figure 1:**
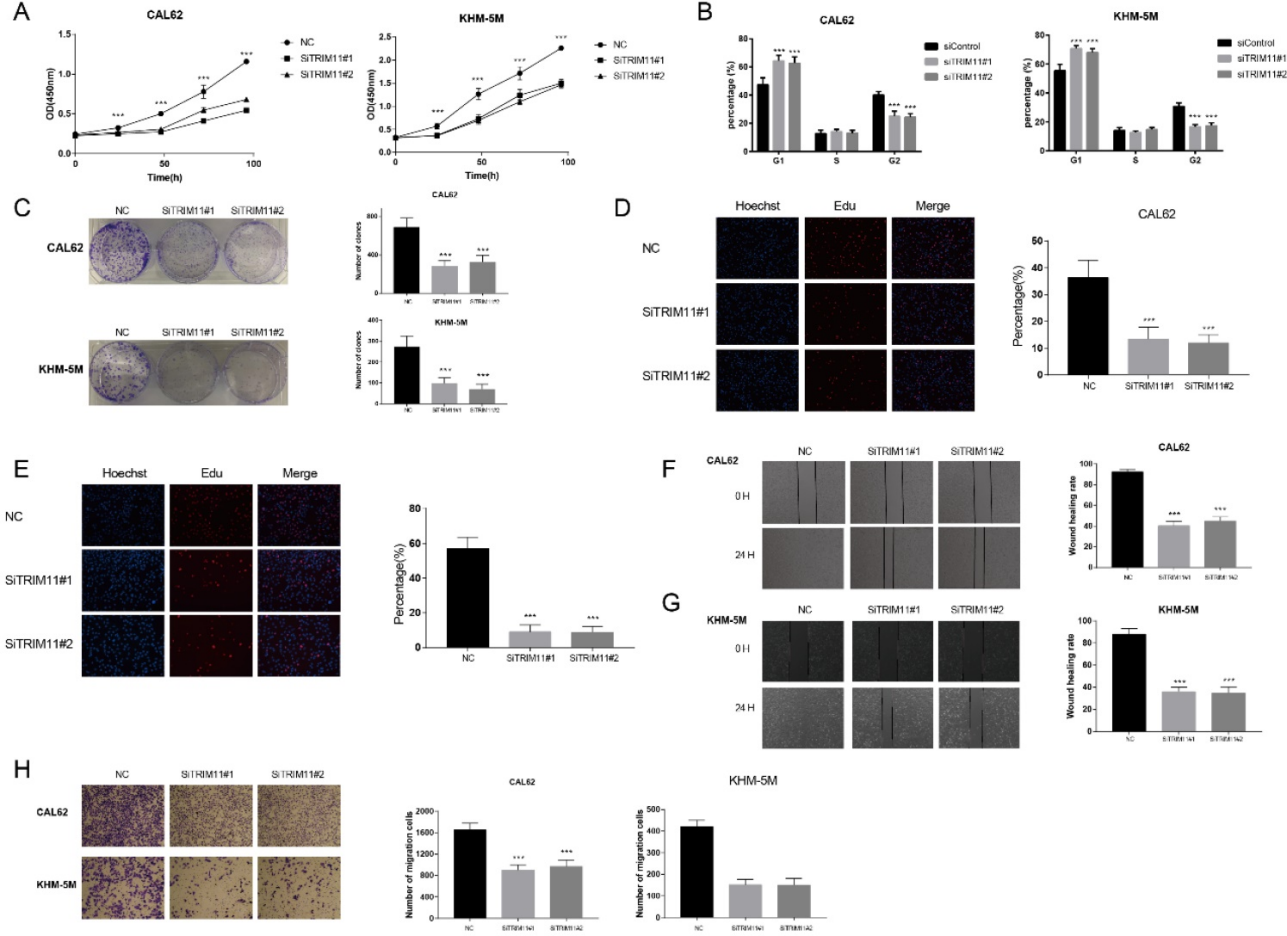
**TRIM11 depletion inhibits anaplastic thyroid cancer cell proliferation and migration.** (A). TRIM11 depletion inhibits the cell proliferation in anaplastic thyroid cancer cells. (B). TRIM11 depletion induces G1 cell cycle arrest in anaplastic thyroid cancer cells. (C). TRIM11 depletion decreases clone formation capability of anaplastic thyroid cancer cells. (D, E). Representative images of EdU assay of anaplastic thyroid cancer cells. (F, G). Wound-healing assay of anaplastic thyroid cancer cells. (H). Transwell migration assay of anaplastic thyroid cancer cells. *, *P* value < 0.05; **,* P* value < 0.01; ***,* P* value < 0.001; ****,* P* value < 0.0001.

We first tested TRIM11 expression level in four kinds of cell lines, including two kinds of papillary thyroid cancer cell lines (B-cpap and TPC-1), two kinds of follicular thyroid cancer cell lines (FTC113 and FTC238), three kinds of ATC cell lines (CAL62, KHM-5M, and 8505C) and a normal thyroid epithelial cell line (Nthy-ori3-1). Our results indicated that mRNA level of TRIM11 was relatively higher in ATC cell lines (Figure S1). Then we chose two ATC cell lines, CAL62 and KHM-5M to investigate the potential functions of TRIM11 in ATC. TRIM11 silence significantly decreased cell proliferation and inhibited G1-S phase transition in both the two cell lines (Figure 1A, B). Knockdown of TRIM11 also decreased the clone formation of CAL62 and KHM-5M cells (Figure 1C). Consistently, siRNA-mediated TRIM11 depletion significantly inhibited DNA synthesis as evaluated by Edu incorporation assay (Figure 1D, E). Wound-healing and transwell assays indicated that depletion of TRIM11 dramatically decreased the migration of CAL62 and KHM-5M cells (Figure 1F-H).

### TRIM11 depletion inhibits Hippo signaling pathway activity

**Figure 2:**
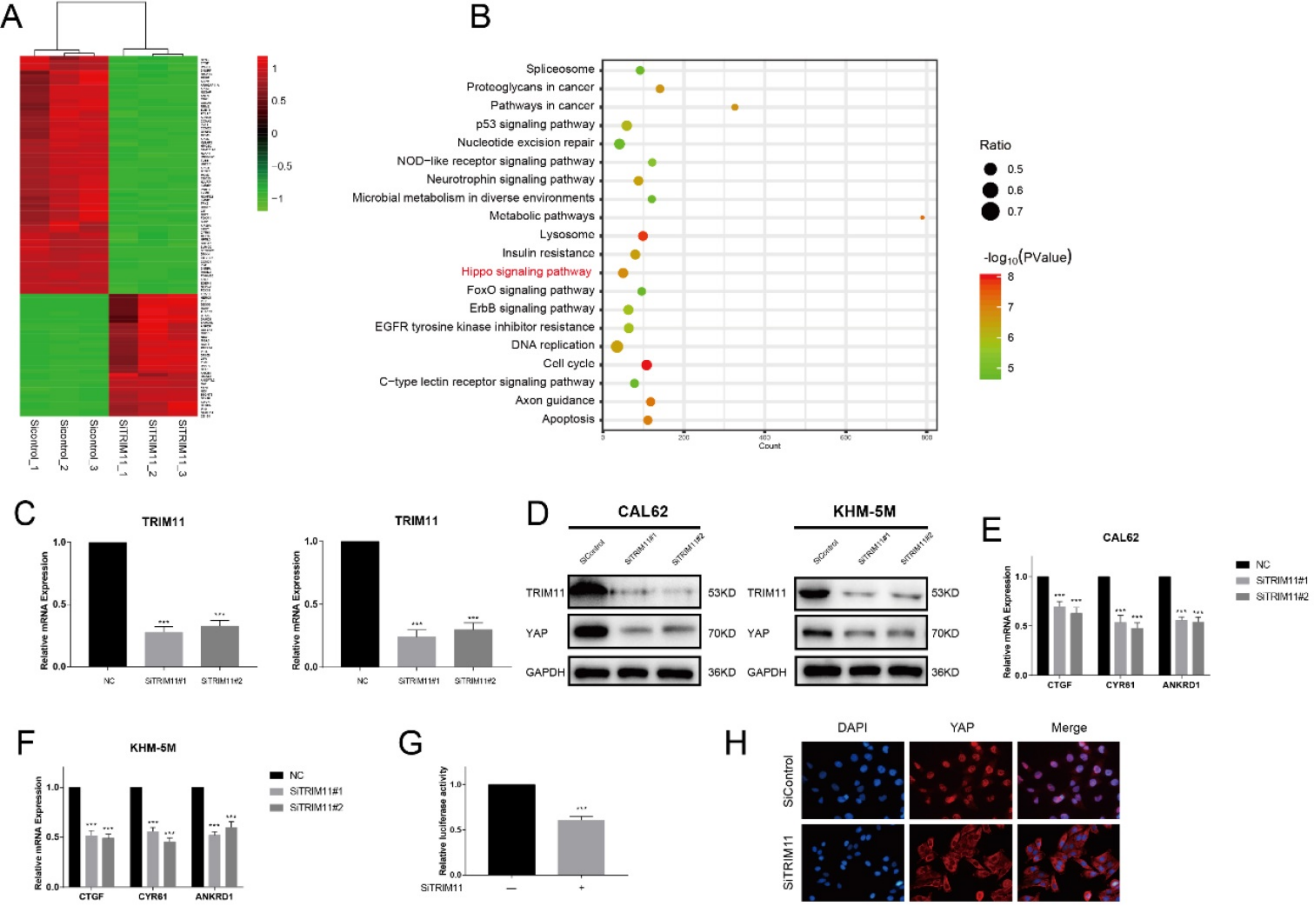
** TRIM11 depletion decreases Hippo signaling activity in anaplastic thyroid cancer cells.** (A). Heatmap of top 100 differentially expressed genes. (B). Pathway enrichment of differentially expressed genes. (C). TRIM11 depletion effect by two different siRNA oligos. Anaplastic thyroid cancer cells were transfected with two independent TRIM11 siRNAs or siControl. After 48 h, TRIM11 mRNA levels were determined by qRT-PCR. 36B4 was used as internal control. (D). TRIM11 depletion decreases YAP protein level. (E, F). TRIM11 depletion decreases YAP target genes using two different siRNA oligos. (G). TRIM11 depletion affects YAP-luciferase activity. CAL62 cells were transfected with SiTRIM11 or SiControl together with YAP-luciferase reporter plasmid. Luciferase activity was measured 48 h after transfection. (H).Immunofluorescence of YAP in KHM-5M cells. *, *P* value < 0.05; **, *P* value < 0.01; ***,* P* value < 0.001.

We next silenced TRIM11 expression in CAL62 cells and examined transcriptional profiles by RNA-seq to further approach the function of TRIM11 in ATC. Compared with the siControl group, YAP target genes (CTGF, CYR61 and ANKRD1) were significantly decreased in SiTRIM11 group, and we noticed that differentially expressed genes were mainly enriched in Hippo signaling pathway (Figure 2A, B). Since YAP is the key effector of Hippo pathway, we then evaluated YAP protein levels in CAL62 and KHM-5M cells using two non-overlapping siRNA targeting TRIM11(Figure 2C). Our results showed that TRIM11 depletion significantly decreased YAP protein level as well as its target gene expression (Figure 2D-F). In agreement, YAP-luciferase reporter activity was strongly suppressed by TRIM11 depletion (Figure 2G). Interestingly, YAP translocated from nuclei to the cytosol after TRIM11 depletion (Figure 2H).

### TRIM11 associates with YAP and increases YAP stability

**Figure 3:**
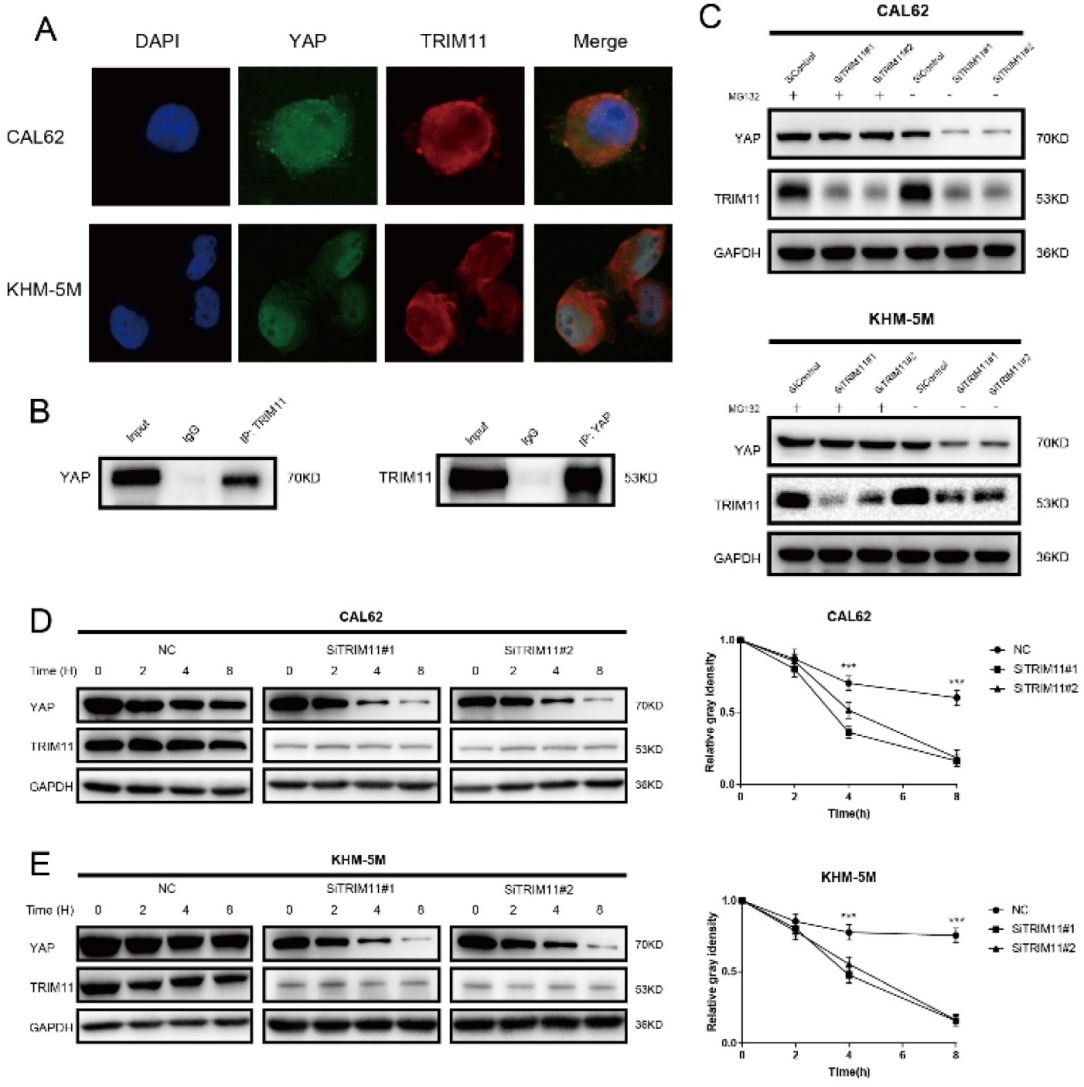
** TRIM11 associates with YAP and increases its stability.** (A) An immunofluorescence assay demonstrated that TRIM11 and YAP at least partially colocalized in CAL62 and KHM-5M cells. (B) Co-IP assay reveals association between endogenous TRIM11 and YAP in CAL62 cells. CAL62 cells were harvested with RIPA lysis buffer. Co-IP was performed using antibody as indicated. (C) In the presence of the proteasome inhibitor MG132, depletion of TRIM11 did not further decrease YAP protein levels. Anaplastic thyroid cancer cells were transfected with siTRIM11 or siControl. After 48 h, cells were treated with 10 nM MG132/vehicle for 6 h, cell lysates were prepared for western blot analysis. (D, E) TRIM11 depletion decreases YAP half-life in anaplastic thyroid cancer cells. Anaplastic thyroid cancer cells were transfected with siTRIM11 or siControl. After 48 h, cells were treated with 100 µM cycloheximide for indicated times. Cell lysates were prepared for western blot analysis.

Results of immunostaining indicated that YAP and TRIM11 were located in both cytoplasm and nucleus (Figure 3A). Endogenous TRIM11 and YAP proteins were co-immunoprecipitated from lysates of CAL62 cells (Figure 3B). GST-pull-down assay showed that TRIM11 interacted with YAP *in vitro* (Figure S2). Depletion of TRIM11 dramatically decreased YAP protein level, while in the presence of the proteasome inhibitor MG132, TRIM11 depletion could not downregulate YAP protein level (Figure 3C). To prove that TRIM11 regulates YAP stability, we treated ATC cells using the protein synthesis inhibitor cycloheximide, the half time of YAP was markedly shortened in ATC cells depleted with TRIM11 (Figure 3D, E).

### Mapping of the binding region between TRIM11 and YAP

**Figure 4:**
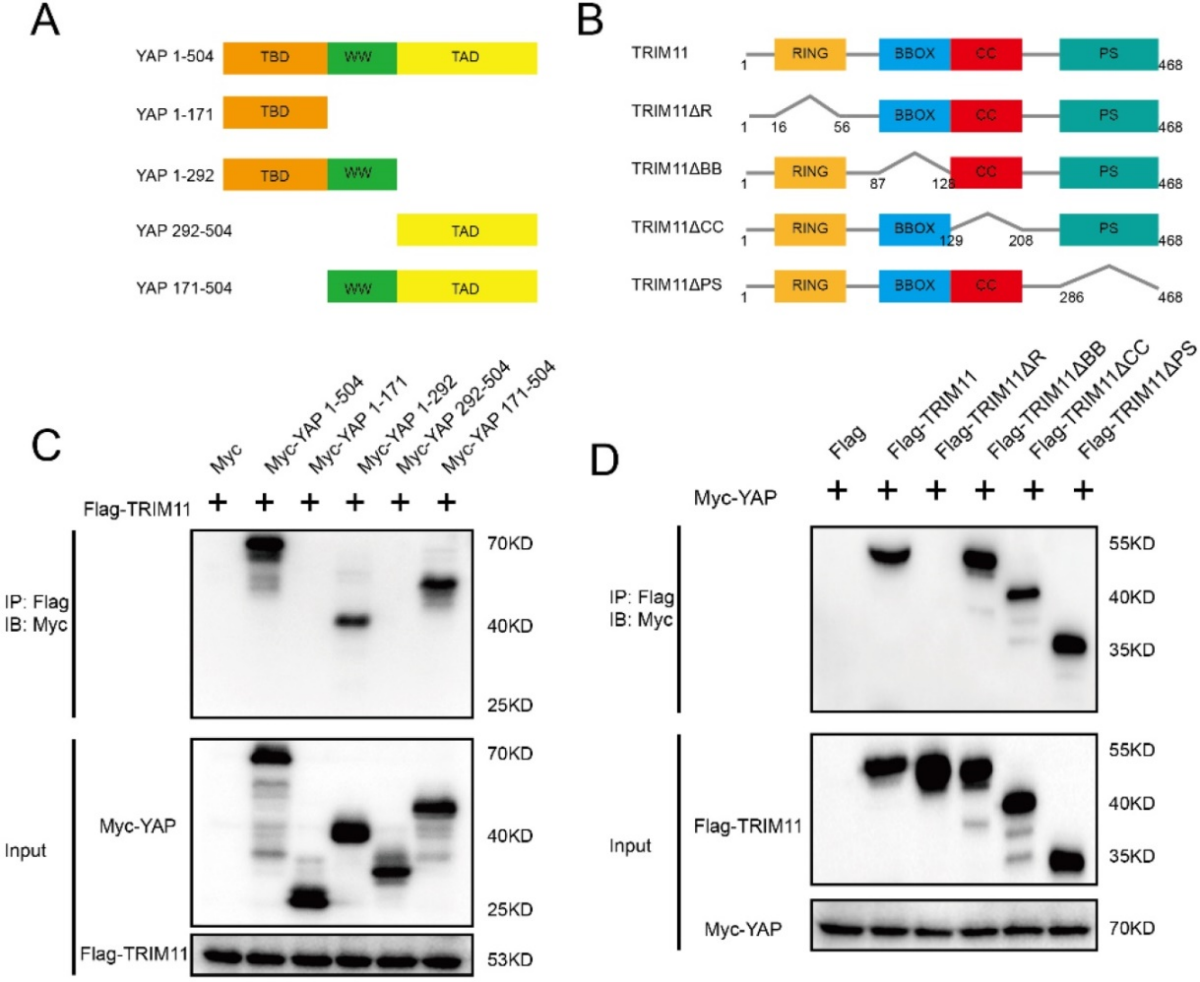
** TRIM11 associates with YAP WW domain through its RING domain.** (A, B). YAP and TRIM11 domain structure and deletion mutants used in the study. (C). TRIM11 interacts with YAP through its WW domain. HEK293 cells were transfected with 2 µg Flag-TRIM11 together with Myc-YAP alpha full length or mutants. After 24 h, cells were harvested with NP-40 lysis buffer. Co-IP was performed using Flag antibody. The possible interacted YAP domains were detected by Myc antibody. (D). RING domain is required for TRIM11 to interaction with YAP. HEK293 cells were transfected with 2 µg Myc-YAP alpha together with Flag-TRIM11 full length or mutants. After 24 h, cells were harvested with NP-40 lysis buffer. Co-IP was performed using Myc antibody. The possible interacted TRIM11 domains were detected by Flag antibody.

YAP has three functional domains: one TEAD transcription factor-binding domain (TBD) mediating most of the interaction with TEAD transcription factors; one (YAP-WW1) or two (YAP-WW1 and YAP-WW2) WW domains, which are abundant and versatile protein-protein interaction modules that recognize proline-rich motifs; and one trans-activation domain (TAD) 33. The deletion mutants of YAP were constructed as follows: ΔTBD (YAP 171-504), ΔTAD (YAP 1-292), ΔTBD + ΔWW (YAP 292-504), and ΔWW + ΔTAD (YAP 1-171) (Figure 4A). TRIM11 is characterized by a RING finger domain, two B-box domains, a coiled-coil domain, and a C-terminal PRY-SPRY (PS) motif. TRIM11 deletion mutants lacking each of the individual domains (ΔR, ΔBB, ΔCC and ΔPS) were constructed to assess the domain(s) of TRIM11 that mediates the association with YAP (Figure 4B). Co-IP assay demonstrated that the RING domain of TRIM11 is necessary for the interaction with YAP WW domain (Figure 4C, D).

### TRIM11 stabilizes YAP possibly via mono-ubiquitination

Ubiquitination assay was performed using a series of ubiquitin mutants. The results indicated TRIM11 dramatically increased the mono-ubiquitination of YAP while inhibited K11- and K48-linked polyubiquitination of YAP (Figure S3). To further assess the functional domain of TRIM11 which modulates YAP ubiquitination, TRIM11 and its deletion mutants were transfected into HEK293T cells together with YAP. TRIM11-ΔR could not promote mono-ubiquitination and inhibit K11- and K48-linked polyubiquitination on YAP protein. However, TRIM11-ΔBB, ΔCC and -ΔPS retained this ability (Figure S4).

**Figure 5:**
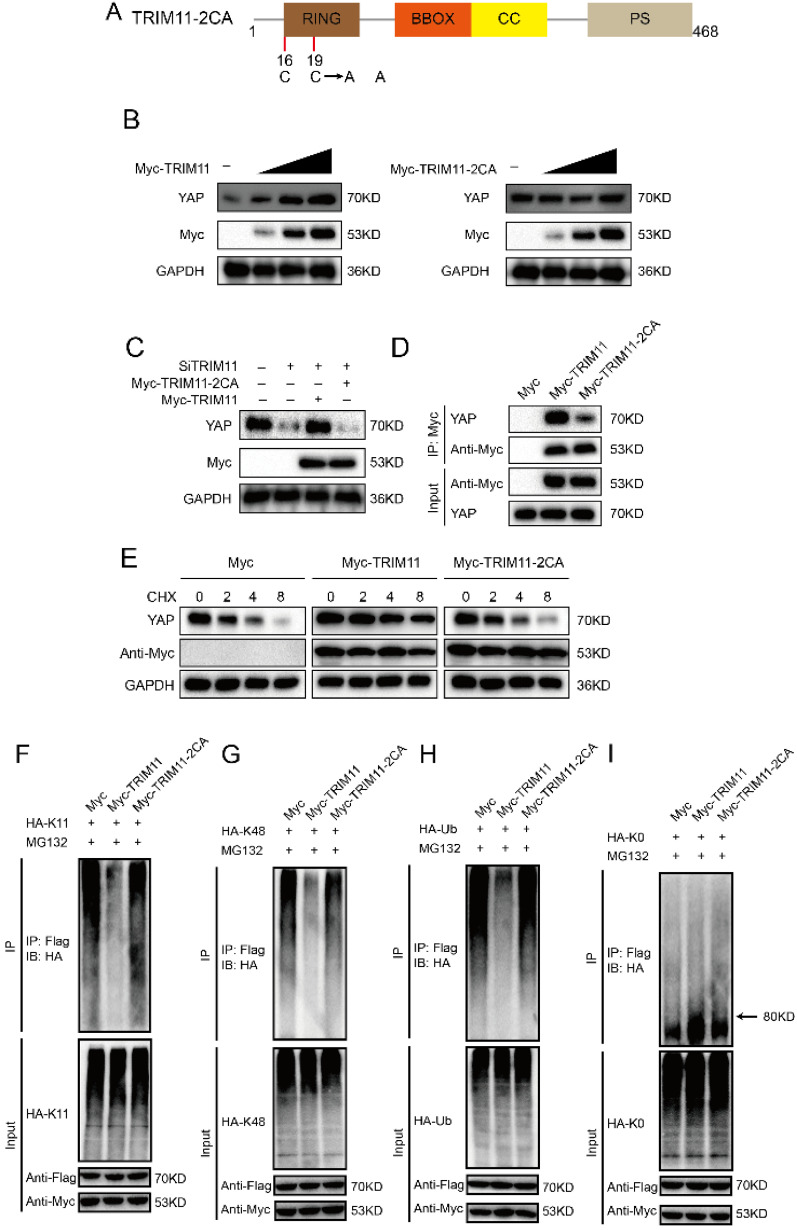
** TRIM11 stabilizes YAP though its RING domain.** (A) Schematic diagram of TRIM11-2CA. (B) Increasing amounts of TRIM11 WT or 2CA were transfected into CAL62 cells and YAP expression was detected. (C). TRIM11 WT or 2CA was introduced into CAL62 cells together with TRIM11 siRNA. YAP levels were measured. (B). Interaction of Myc-TRIM11-2CA proteins with endogenous YAP in CAL62 cells was analyzed by co-IP assay. (E). HEK293 cells transfected with the indicated plasmids were treated with 100 µM cycloheximide, and collected at the indicated times for western blot. (F-I) TRIM11-2CA does not modulate poly/mono-ubiquitination of YAP protein.

Since RING domain is essential for TRIM11 function, we change two conserved Cys resides (Cys16 and 19) in the RING domain to Ala (TRIM11-2CA), which are involved in Zn2+ binding function (Figure 5A). Ectopic expression of wildtype TRIM11, but not TRIM11-2CA, resulted in YAP elevation in a dose-dependent manner (Figure 5B). The decrease of YAP induced by TRIM11 depletion could be reversed by overexpression of wildtype TRIM11, while TRIM11-2CA had no such effect (Figure 5C). In addition, TRIM11-2CA had a significantly reduced ability to associate with YAP and could not increase YAP stability (Figure 5D, E). Ubiquitination assay indicated that TRIM11-2CA did not promote mono-ubiquitination on YAP and lost the ability to inhibit K11- and K48-linked polyubiquitination on YAP (Figure 5F-I). Taken together, our results indicate that the RING domain TRIM11 modulates YAP stability through a direct protein-protein interaction that involves the WW domain of YAP.

### TRIM11 promotes anaplastic thyroid cancer progression via YAP

**Figure 6:**
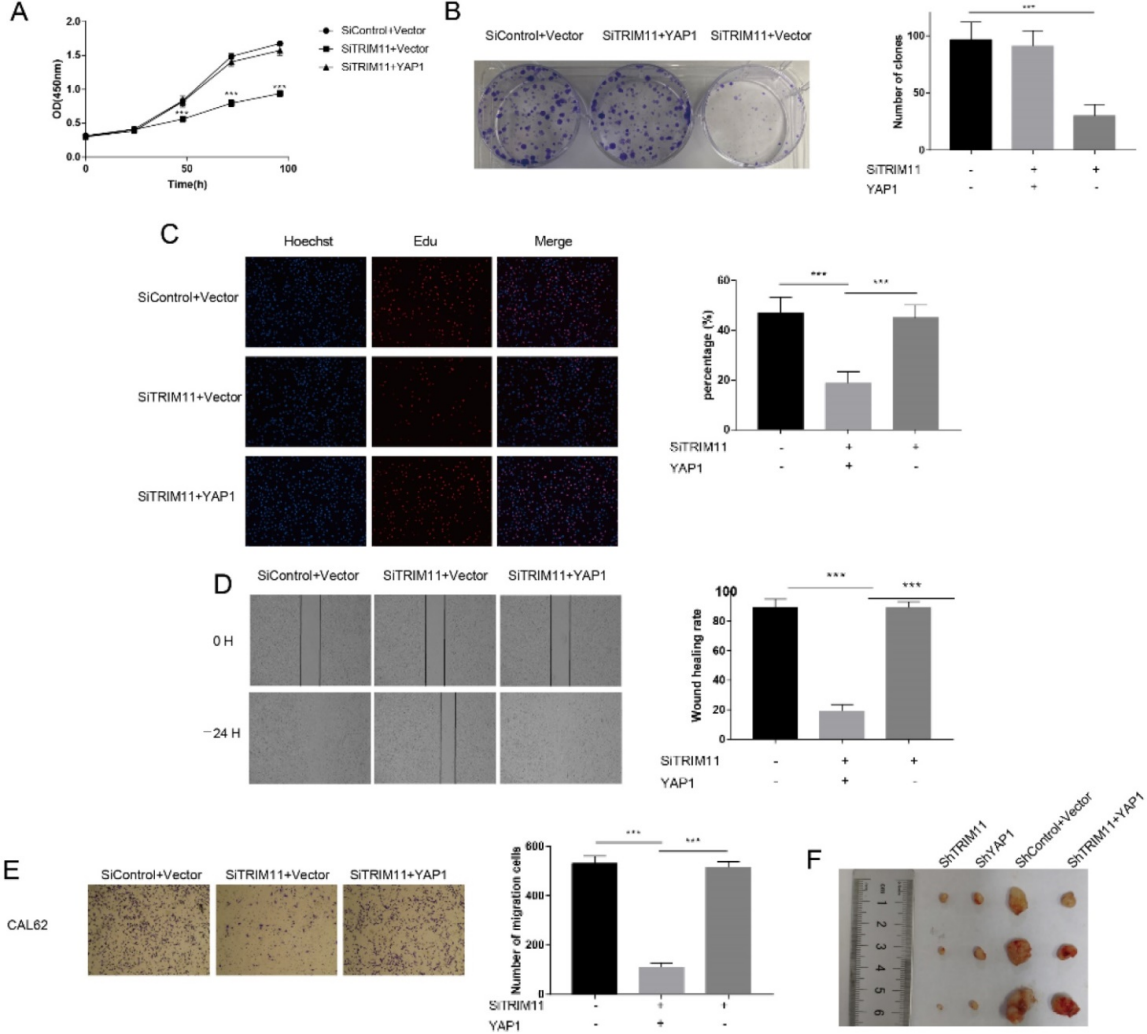
** Increased YAP expression recovered the effect of TRIM11 depletion.** (A) Cell proliferation assay of CAL62. (B) Clone formation assay of CAL62. (C). Representative images of EdU assay of CAL62. (D) Wound-healing assay of CAL62. (E) Transwell migration assay of CAL62. (F) Overexpression of YAP in TRIM11-knockdown cells partly recovered tumor growth *in vivo*. *, *P* value < 0.05; **,* P* value < 0.01; ***,* P* value < 0.001.

The results identified above suggested that TRIM11 might exert its function through YAP. The oncogenic function of YAP has been validated in most solid tumors. However, its role in ATC has not been revealed. We then depleted YAP in CAL62 and KHM-5M cells. YAP depletion dramatically inhibited cell proliferation and migration (Figure S5). To further verify whether TRIM11 promoted cell proliferation and migration in a YAP-dependent manner, we overexpressed YAP in TRIM11 depletion cells and performed a rescue experiment. YAP overexpression largely recovered the suppressive effects induced by TRIM11 depletion (Figure 6A-E), suggesting that TRIM11 may promote ATC proliferation and migration by regulating YAP. We then used xenograft mice models to further investigate the role of TRIM11 in tumor growth. Our data indicated that depletion of TRIM11 or YAP by lentivirus-based shRNA inhibited tumor growth, while the overexpression of YAP in TRIM11-knockdown cells partly recovered tumor growth (Figure 6F).

### TRIM11 regulates response of anaplastic cancer cells to chemotherapy through YAP

Doxorubicin is commonly used for ATC. The activity of YAP is associated with chemoresistance in various types of malignancies including ovarian cancer, breast cancer, and hepatocellular carcinoma. Since YAP is stabilized by TRIM11, we then examined whether inhibition of TRIM11 affects cell response to doxorubicin. Cells treated with TRIM11 siRNA or YAP siRNA were more sensitive to doxorubicin, and the effects induced by TRIM11 depletion could be abolished by YAP overexpression (Figure S6).

### TRIM11 is a direct transcriptional target of SOX13

**Figure 7:**
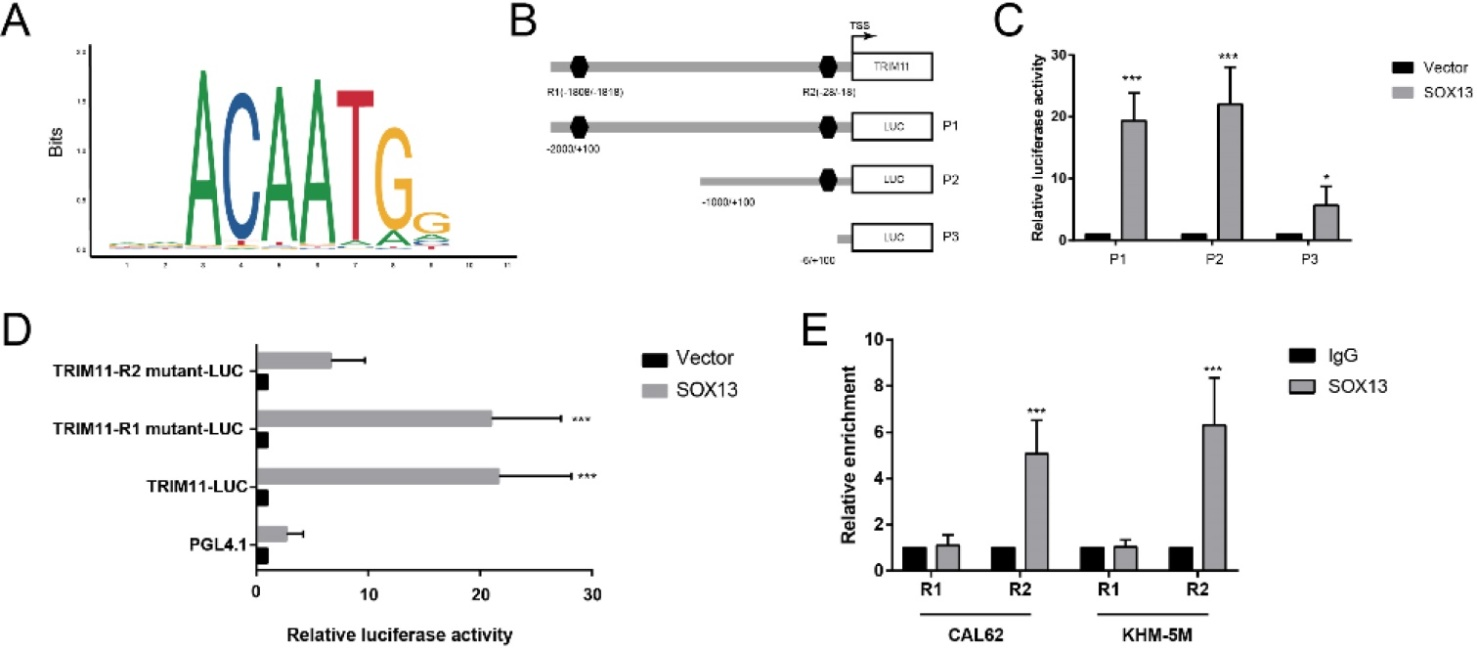
** SOX13 binds directly to the TRIM11 promoter.** (A) The binding site of SOX13 provided by the JASPAR database. (B). A diagram shows the relative position of full-length and fragments of TRIM11 promoter reporters. (C, D) Serially truncated and mutated TRIM11 promoter constructs were transfected together with empty vector or SOX13 overexpression plasmids into CAL62 cells. Then a luciferase reporter assay was utilized. (E). A ChIP assay demonstrated the direct interactions between SOX13 and the TRIM11 promoter in the indicated anaplastic thyroid cancer cell lines. *, P value < 0.05; **,* P* value < 0.01; ***,* P* value < 0.001.

Regulation of TRIM11 transcription is still not well understood. We used PROMO database (http://alggen.lsi.upc.es/cgi-bin/promo_v3/promo/promoinit.cgi?dirDB=TF_8.3) and JASPAR database (http://jaspar.genereg.net/) to analyze the human TRIM11-promoter nucleotide sequence, and 2 putative binding motifs were found respectively (Figure 7A, B). We then constructed and transfected a series of luciferase reporter plasmids flanked with truncated or mutated TRIM11 promoter sequences into CAL62 cells for a luciferase reporter assay. Serial deletion suggested that the binding site R2 (-28 to -18 bp) was essential to SOX13-induced expression of luciferase reporter. In addition, site-directed mutation of TRIM11 also indicated that the binding site R2 was dispensable for SOX13 binding and the transcription of luciferase reporter (Figure 7 C, D). Furthermore, the chromatin immunoprecipitation (ChIP) assay demonstrated that SOX13 directly bound to the putative sites of TRIM11 in ATC cell lines (Figure 7E). These findings demonstrated that TRIM11 was direct transcriptional targets of SOX13.

## Discussion

TRIM11 is a member of Tripartite Motif Containing (TRIM) proteins characterized by an N-terminal TRIM/RBCC motif. This motif contains a RING domain, 1 or 2 B-box motifs and a coiled-coil region (RBCC). Besides the N-terminal RBCC domain, TRIM11 contains a C-terminal PRY-SPRY (PS) motif 34. In lung cancer, TRIM11 is reported to promote cell proliferation and invasion via activating the PI3K/AJT pathway 29. Consistently, TRIM11 is a component in PHLPP1/AKT signaling pathway in human chordoma cells. Expression of TRIM11 is upregulated in chordomas tissues and promotes chordoma cells proliferation 35. In hepatocellular carcinoma, TRIM11 exerts its oncogenic effects through the inhibition of P53 36. In addition, TRIM11 can suppress the anti-tumor effect of proteotoxic stress drugs through cooperating with HSF1 37. Thus, identifying novel substrate is essential for understanding TRIM11 biology and its implication in tumorigenesis and drug resistance.

The present study indicates YAP activation is important in ATC. YAP depletion dramatically inhibited cell proliferation and migration of ATC. YAP is the evolutionarily conserved key element of the Hippo pathway, which mediates its function through regulation gene transcription 38. The Hippo pathway is a newly identified pathway which is highly conserved in mammals. This pathway is known to regulate organ size and tissue growth through a delicate balance between cell proliferation and apoptosis 39. Accumulating studies indicate the prominent role of the Hippo pathway in tumorigenesis. The dysregulation of this pathway has been observed in various types of cancer: including breast, lung, liver, colon, and others 40, 41. Hippo pathway may have an important role in the initiation and progression of cancer, YAP activation results in cell transformation and tumor development 42. Elevated YAP expression helps cells to escape contact inhibition and promotes cell survival, proliferation, migration, and invasion 40. The activity of YAP is mainly regulated by the MST1/2-Lats1/2 kinase cascade. LATS1/2 directly phosphorylates YAP on multiple sites, resulting in the interaction with 14-3-3 protein and cytoplasmic retention 43. Besides the mechanisms regulating its phosphorylation and localization, YAP can be controlled by other post-translational modification such as ubiquitination. For instance, Fbxw7 regulates YAP protein stability by targeting YAP for ubiquitination and proteasomal degradation in hepatocellular carcinoma 44; SHARPIN and RNF187 promote YAP degradation via inducing YAP K48-dependent poly-ubiquitination 45, 46. Deubiquitinating enzymes (DUBs) also regulate the stability of YAP protein in human cancers. In breast cancer, USP9X deubiquitinates and stabilizes YAP to promote breast cancer cell proliferation and chemoresistance to therapeutic drugs 47. USP47 functions as a DUB for YAP in colorectal cancer, USP47 elevation leads to stabilization of YAP and promotes colorectal cancer cell proliferation 48. DUB3 regulates the protein stability of multiple components of Hippo pathway, including the LATS kinases, the E3 ligase ITCH and the AMOT family proteins, which in turn induces YAP turnover 49. Thus, Ubiquitination and de-ubiquitination are important for maintaining the function of Hippo pathway. In this study, we observed that YAP can be modified by both mono-and polyubiquitination, and mono-ubiquitination was more likely to confer the stability of YAP by inhibiting its polyubiquitination and degradation.

ATC is one of the most aggressive and virulent solid tumors. There exists no effective or standard therapy for the treatment of ATC. Therefore, it is an urgent issue to explore the underlying molecule mechanisms involved in the initiation and progression of ATC. More novel candidate targets are needed to improve the treatment decisions. Here, we found TRIM11 may be a potential therapeutic target of ATC. In the present study, we examined the biological function of TRIM11 using ATC cell lines. TRIM11 depletion significantly inhibited cell proliferation and induced G1 phase arrest. Transwell assay demonstrated that the capability of migration was decreased upon TRIM11 silence. And the suppression effects induced by TRIM11 depletion could be reversed by YAP overexpression. Global gene analysis based on RNA-seq indicated that TRIM11 depletion significantly suppressed Hippo pathway activity. In addition, TRIM11 depletion significantly decreased YAP protein level as well YAP-luciferase reporter gene activity. Since TRIM11 is an E3 ubiquitin ligase, we then investigated whether TRIM11 could directly interact with YAP and regulate its protein stability. We performed co-ip analysis to identify the association between YAP and TRIM11, the results demonstrated that RING domain of TRIM11 is essential for the interaction with the WW domain of YAP. Upon inhibition of protein synthesis by cycloheximide, YAP protein half time was significantly decreased in CAL62 and KHM-5M cells depleted with TRIM11. To further analysis the underlying mechanisms, a series of mutant ubiquitin were used to identify the linkage of ubiquitin chain. We observed that TRIM11 could dramatically increase the mono-ubiquitination of YAP while inhibit K11- and K48-linked polyubiquitination on YAP protein. Furthermore, TRIM11-2CA lost the ability to promote YAP mono-ubiquitination and inhibit K11- and K48-linked YAP polyubiquitination.

SOX family proteins fulfill their roles as transcription factors by controlling the expression of dozens of target genes at the transcription level. Accumulating evidence has revealed the involvement of different Sox proteins in tumor progression and metastasis, while few studies have revealed the biological function of SOX13 50. Recent studies indicated that SOX13 functions as an oncogene and promotes the proliferation and metastasis of gastric and colorectal cancer 51, 52. In hepatocellular carcinoma, SOX13 promotes cell invasion, migration, and epithelial-to-mesenchymal transition through activating Twist1 transcription 53. In the present study, TRIM11 promoter analysis revealed that SOX13 activated TRIM11 transcription by binding to 18 bp upstream from the transcription start site in the promoter of TRIM11.

**Figure 8:**
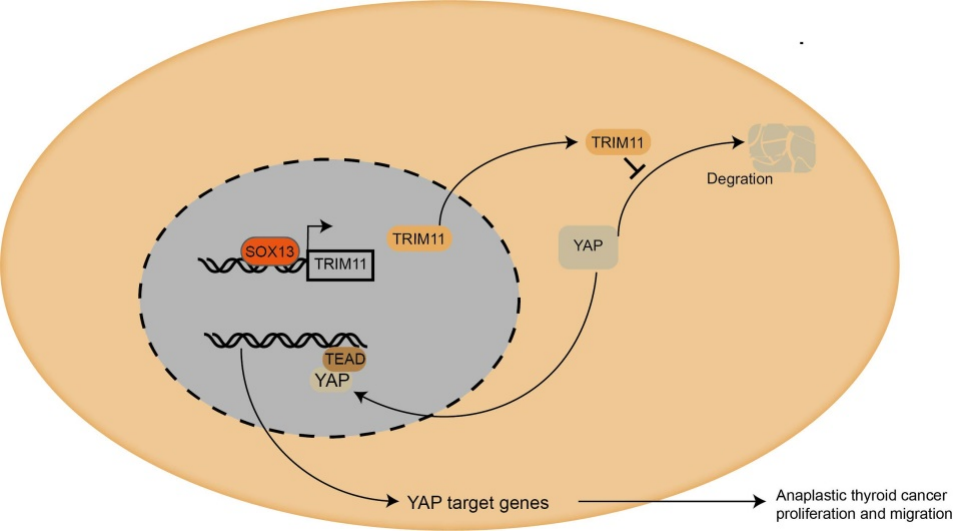
** Mechanism diagram.** Our study demonstrated that TRIM11could promote anaplastic thyroid cancer cells proliferation and migration both *in vitro* and *in vivo*. Furthermore, TRIM11 could increase YAP protein stability in ATC cells. SOX13 could activate transcription of TRIM11 in ATC cells by binding to the promoter region.

Taken together, we proposed a SOX13/TRIM11/YAP axis in the progression of ATC. TRIM11, as a transcriptional target of SOX13, regulates the stability of YAP through a direct protein-protein interaction that involves the WW domain of YAP and the RING domain of TRIM11 (Figure 8). And the stabilization of YAP mediated by TRIM11 may depend on its mono-ubiquitination modification. Thus, targeting TRIM11 could be a potential strategy or drug target for anaplastic thyroid cancer.

## Supplementary Material

Supplementary figures and tables.
