# Supplementary material for: SOX13/TRIM11/YAP axis promotes the proliferation, migration and chemoresistance of anaplastic thyroid cancer
Source: Int J Biol Sci. 2021 Jan 1;17(2):417–29. doi: 10.7150/ijbs.54194 (PMC7893578; doi:10.7150/ijbs.54194)
Supplement: Supplementary file 1 — Supplementary figures and tables. [file ijbsv17p0417s1.pdf]

Supplementary legends

Figure S1. Relative expression level of TRIM11 thyroid cancer cell lines.

Figure S2. Pulldown assay reveals direct interaction between TRIM11 and YAP. CAL62 cells transfected with Flag-TRIM11 were lysed and lysates incubated with GST-YAP or GST protein. The interacted TRIM11 was detected via western blot.

Figure S3. TRIM11 increases the mono-ubiquitinated YAP while decreases K11- and K48-dependent polyubiquitination on YAP protein. (A-I). HEK293 cells were transfected with 2 μg Myc-YAP plasmid, 0.5 μg Flag-TRIM11 plasmids, and 0.5 μg HA-K6, -K11, -K27, -K29, -K33, -K48, -K63, -K0 or -Ub plasmids. The cell extracts were immunoprecipitated with HA antibody. The specific polyubiquitinated and mono-ubiquitinated YAP were detected via western blotting analysis.

Figure S4. TRIM11 increases the mono-ubiquitinated YAP and decreases K11- and K48-dependent polyubiquitination on YAP protein via its RING domain. (A-D). HEK293 cells were transfected with 2 μg Myc-YAP plasmid, 0.5 μg Flag-TRIM11 or its deletion mutant plasmids, and 0.5 μg HA-K11, -K48, -K0 or -Ub plasmids. The cell extracts were immunoprecipitated with HA antibody. The specific polyubiquitinated and mono-ubiquitinated YAP were detected via western blotting analysis.

Figure S5. YAP depletion inhibits anaplastic thyroid cancer cell proliferation and migration. (A). YAP depletion inhibits the cell proliferation in anaplastic thyroid cancer cells. (B, C). Representative images of EdU assay of anaplastic thyroid cancer cells. (D). YAP depletion decreases clone formation capability of anaplastic thyroid cancer cells. (E). Wound-healing assay of anaplastic thyroid cancer cells. (F). Transwell migration assay of anaplastic thyroid cancer cells.

*, *P value < 0.05; **, P value < 0.01; ***, P value < 0.001.*

Figure S6. TRIM11 regulates the anaplastic cancer cells response to chemotherapy. (A-C). CAL62 Cells expressing indicated constructs were treated with doxorubicin, and cell survival was determined. (D-F). KHM-5M Cells expressing indicated constructs were treated with doxorubicin, and cell survival was determined. (G). In vivo xenografts generated from CAL62 cells expressing an empty vector, TRIM11 or YAP-targeting shRNA and treated with doxorubicin.

Figure S1

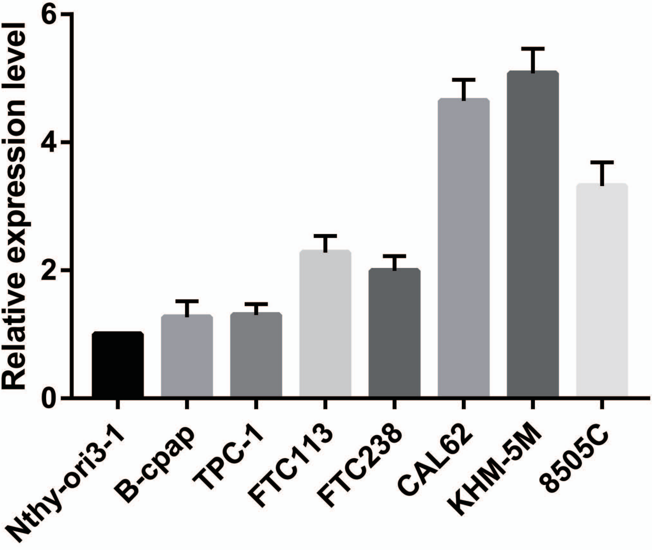

Relative expression level
6
4
2
0
Nthy-ori3-1
B-cpap
TPC-1
FTC113
FTC238
CAL62
KHM-5M
8505C

Figure S2

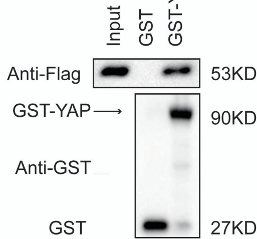
Input
GST
GST-YAP
Anti-Flag
53KD
GST-YAP
90KD
Anti-GST
GST
27KD

Figure S3

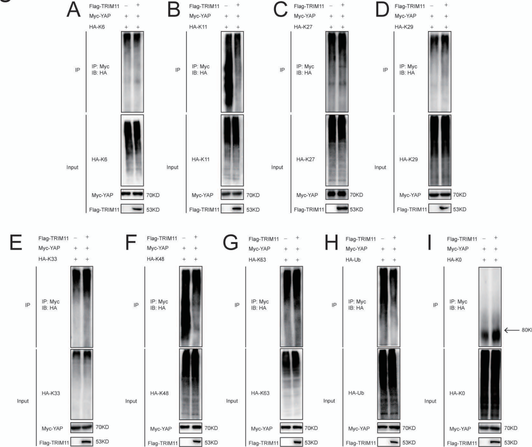
A
B
C
D
E
F
G
H
I
Flag-TRIM11 − +
Myc-YAP + +
HA-K6 + +
HA-K11 + +
HA-K27 + +
HA-K29 + +
HA-K33 + +
HA-K48 + +
HA-K63 + +
HA-Ub + +
HA-K0 + +
IP
IP: Myc
IB: HA
Input
HA-K6
HA-K11
HA-K27
HA-K29
HA-K33
HA-K48
HA-K63
HA-Ub
HA-K0
Myc-YAP
70KD
Flag-TRIM11
53KD
80KD

Figure S4

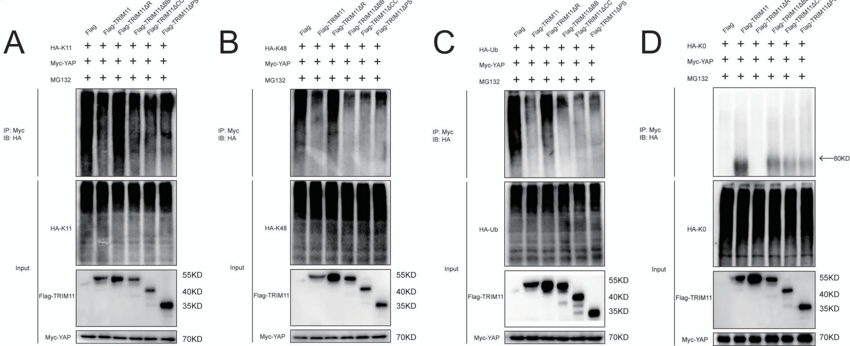
A
B
C
D
Flag
Flag-TRIM11
Flag-TRIM11ΔR
Flag-TRIM11ΔBB
Flag-TRIM11ΔCC
Flag-TRIM11ΔPS
HA-K11
HA-K48
HA-Ub
HA-K0
Myc-YAP
MG132
IP: Myc
IB: HA
Input
Flag-TRIM11
80KD
55KD
40KD
35KD
70KD

Figure S5

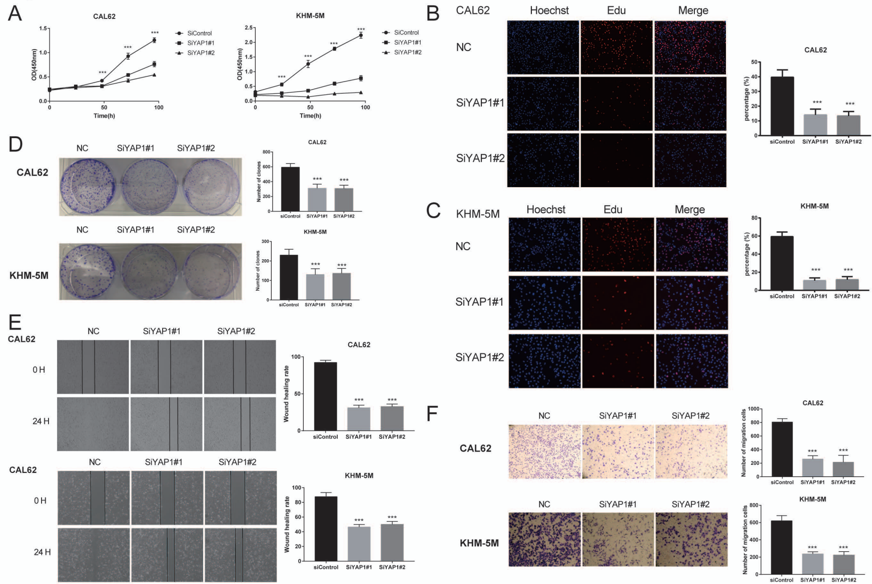
A
CAL62
SiControl
SiYAP1#1
SiYAP1#2
OD(450nm)
Time(h)
KHM-5M
B
CAL62
Hoechst
Edu
Merge
NC
SiYAP1#1
SiYAP1#2
percentage (%)
siControl
C
KHM-5M
D
Number of clones
E
0 H
24 H
Wound healing rate
F
Number of migration cells

Figure S6

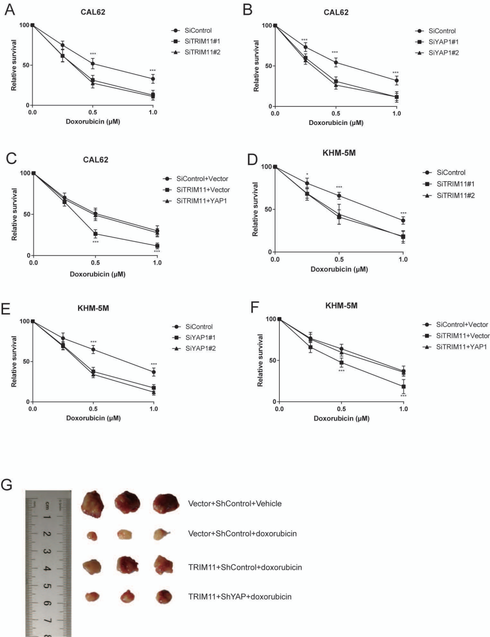

A
CAL62
Relative survival
Doxorubicin (μM)
SiControl
SiTRIM11#1
SiTRIM11#2
B
CAL62
Relative survival
Doxorubicin (μM)
SiControl
SiYAP1#1
SiYAP1#2
C
CAL62
Relative survival
Doxorubicin (μM)
SiControl+Vector
SiTRIM11+Vector
SiTRIM11+YAP1
D
KHM-5M
Relative survival
Doxorubicin (μM)
SiControl
SiTRIM11#1
SiTRIM11#2
E
KHM-5M
Relative survival
Doxorubicin (μM)
SiControl
SiYAP1#1
SiYAP1#2
F
KHM-5M
Relative survival
Doxorubicin (μM)
SiControl+Vector
SiTRIM11+Vector
SiTRIM11+YAP1
G
Vector+ShControl+Vehicle
Vector+ShControl+doxorubicin
TRIM11+ShControl+doxorubicin
TRIM11+ShYAP+doxorubicin
